# The Onstep Method for Inguinal Hernia Repair: Operative Technique and Technical Tips

**DOI:** 10.1155/2016/6935167

**Published:** 2016-06-09

**Authors:** Jacob Rosenberg, Kristoffer Andresen

**Affiliations:** Department of Surgery, Herlev Hospital, University of Copenhagen, Herlev Ringvej 75, 2730 Herlev, Denmark

## Abstract

Inguinal hernia repair is one of the most common surgical procedures and several different surgical techniques are available. The Onstep method is a new promising technique. The technique is simple with a number of straightforward steps. This paper provides a full description of the technique together with tips and tricks to make it easy and without complications.

## 1. Introduction

Inguinal hernias affect millions of people each year [1, 2]. Early on it was acknowledged that there was a need to reinforce the abdominal wall and it has been proven that, in the general treatment of inguinal hernias, there is a need for some kind of mesh to minimize the risk of recurrence [3]. Surgeons are seeking the optimal mesh and location and method of placement. Two methods are currently dominating, one being the open anterior approach, the Lichtenstein repair, and the other being the posterior approach, the laparoscopic repair [4]. Many other methods have been tested and even robot-assisted groin hernia repair has now been reported [5]. The focus now is on debilitating chronic pain that occurs in 0.5–6% of patients [6].

The laparoscopic and the Lichtenstein methods are dominating, probably because of reproducible results and the relative simplicity of the techniques, which allows for surgeons worldwide to learn and teach the techniques. There are, however, still complications related to the Lichtenstein and the laparoscopic techniques and concern regarding cost and learning issues with the laparoscopic technique. For laparoscopic techniques there is a need for endoscopic equipment which increases the costs [7] and the Lichtenstein technique has an increased risk of chronic pain, which may be severe and disabling [8]. Chronic pain after inguinal hernia repair is difficult to treat successfully [9].

There is a need for a simple technique that does not require the same equipment and training as the laparoscopic technique but still results in low risk of chronic pain. One such method seems to be the Onstep technique [10]. The Onstep technique is simple, has a short duration of surgery, and consists of a series of standardized steps. However, in order to ensure that the procedure is conducted the right way, which will also allow for comparison of results across institutions, there is a need for a thorough presentation of the technique. Furthermore, we have found a few tips and tricks that facilitate the procedure.

## 2. The Operative Steps

The patient should be positioned in a supine, flat position and under general anesthesia. For a list of required surgical instruments, see the following.

Materials for the Onstep Procedure are as follows:

 Electrocautery.
 Scissors (mayo and metzenbaum).
 Forceps.
 Langenbeck retractor.
 Rubberband or similar.
 Nonabsorbable suture for the mesh.
 Absorbable suture for fascia closure.
 Onflex-mesh, most often size medium.
 Gauze 30 × 40 cm.
 Saline.

**Figure 1:**
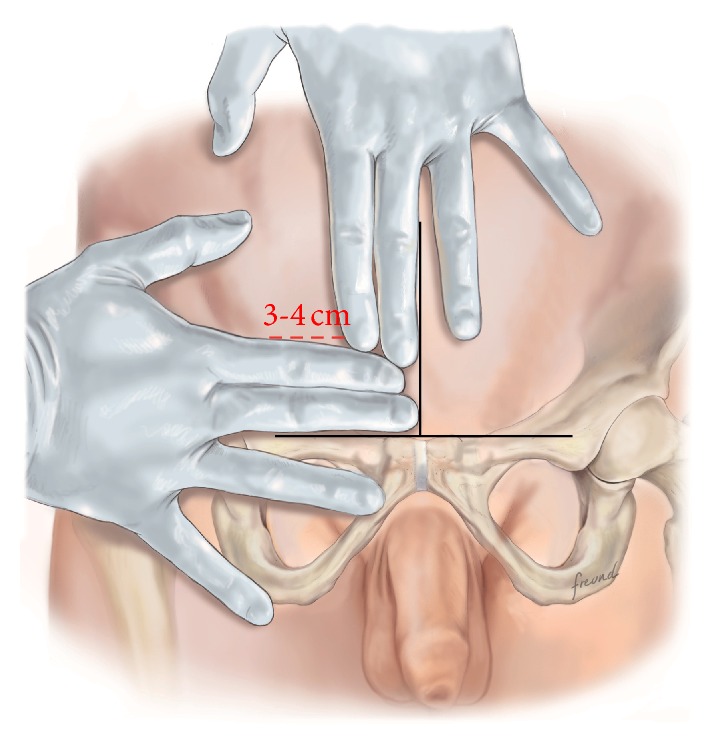
The incision is marked by the index and third fingers as shown.

**Figure 2:**
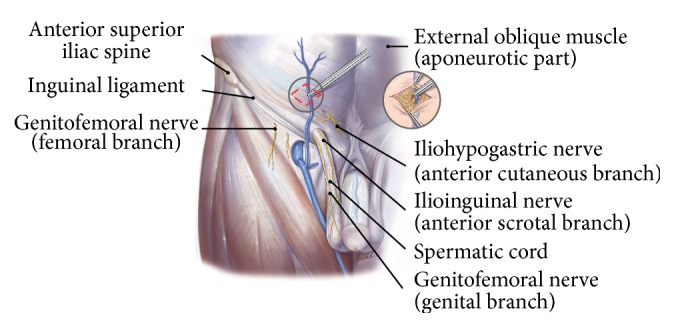
The superficial epigastric vein in the subcutaneous tissue layer is cauterized.

**Figure 3:**
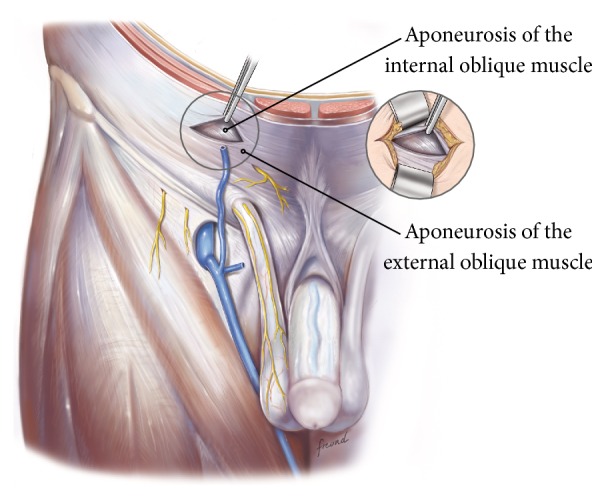
The external aponeurosis is incised to expose the underlying internal aponeurosis.

**Figure 4:**
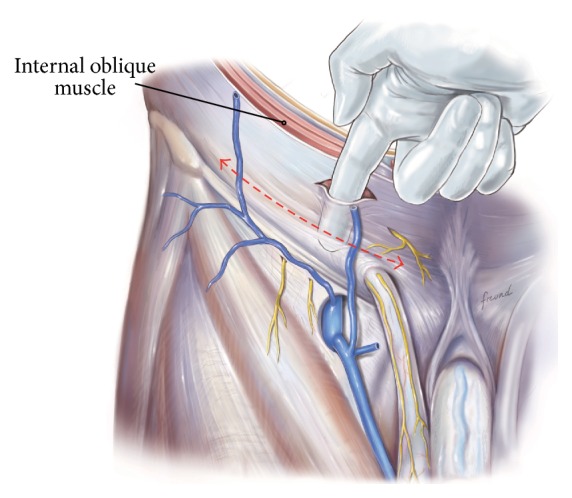
Blunt dissection using the finger will create the necessary surgical space for the operation.

**Figure 5:**
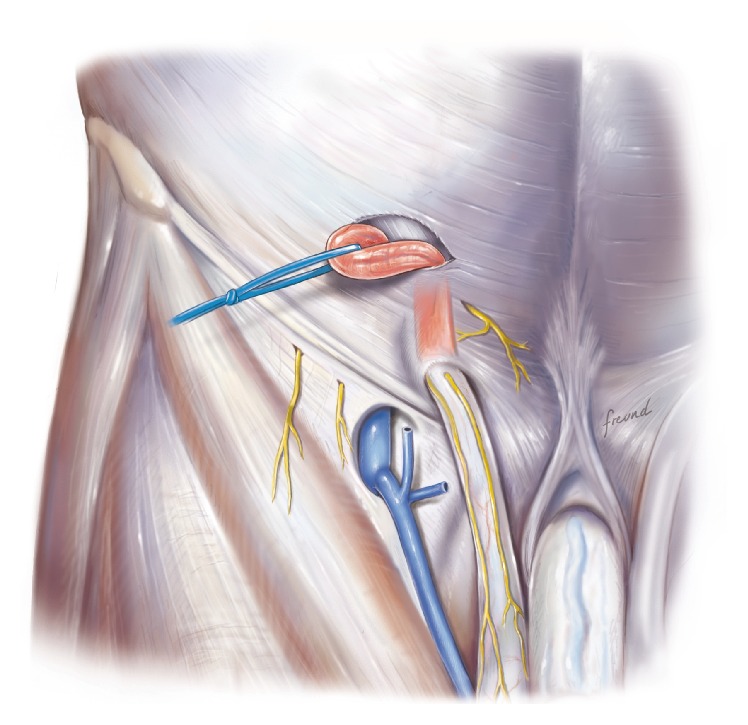
The cord is dissected and lifted out of the incision.

**Figure 6:**
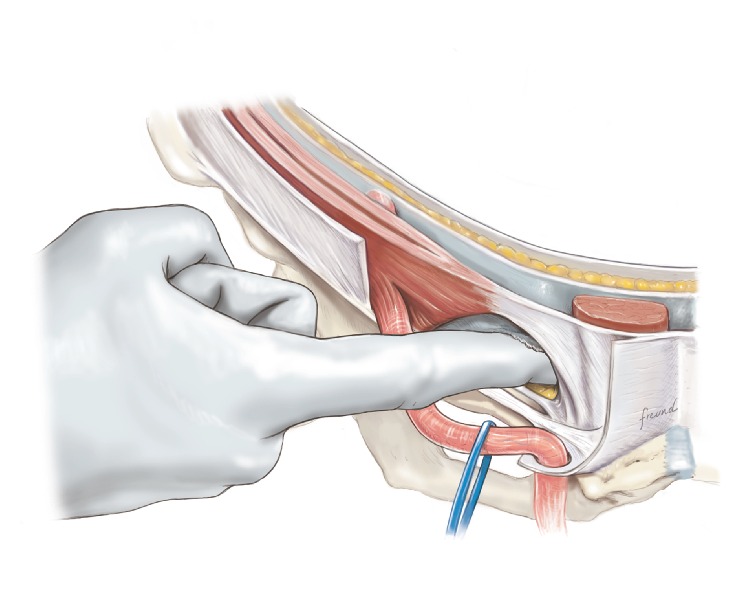
The index finger is used to plunge through the transversalis fascia into the preperitoneal space.

**Figure 7:**
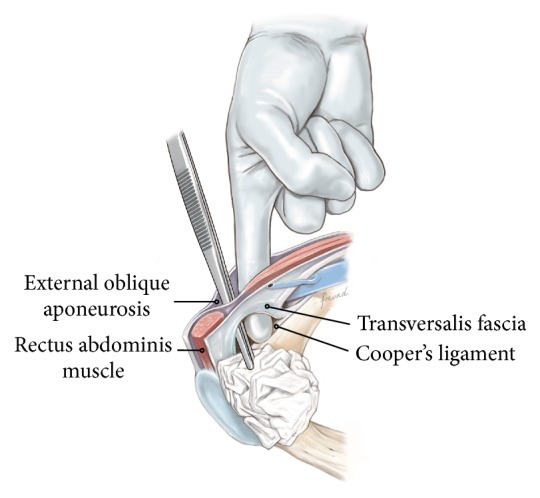
Moist sterile gauze is placed in the preperitoneal space.

**Figure 8:**
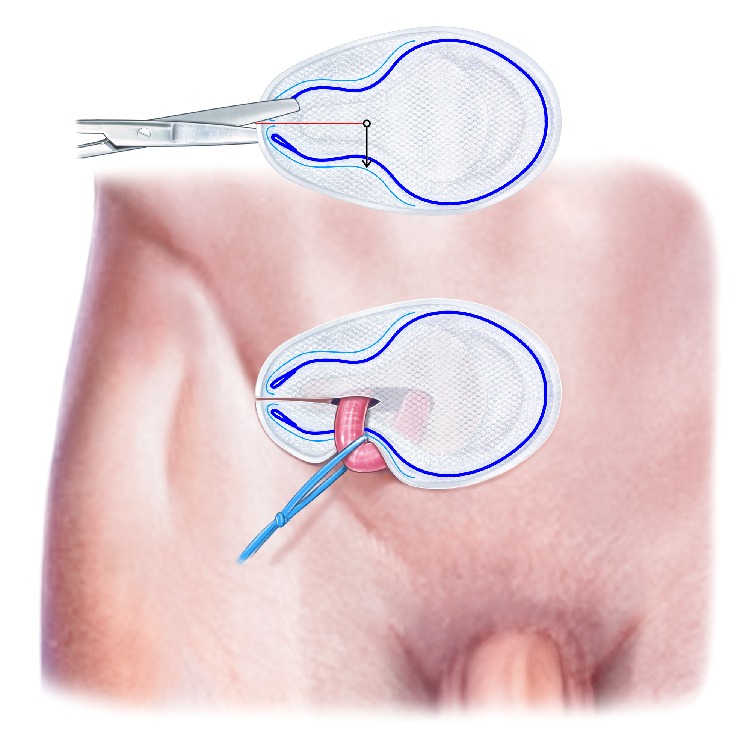
The Onflex mesh is cut and the tails are placed around the spermatic cord.

**Figure 9:**
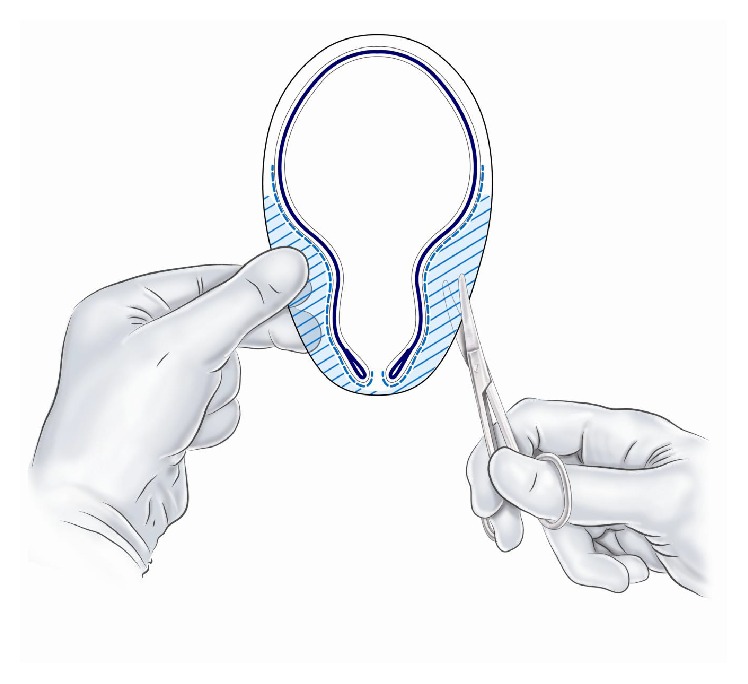
The lateral parts of the Onflex mesh are removed.

**Figure 10:**
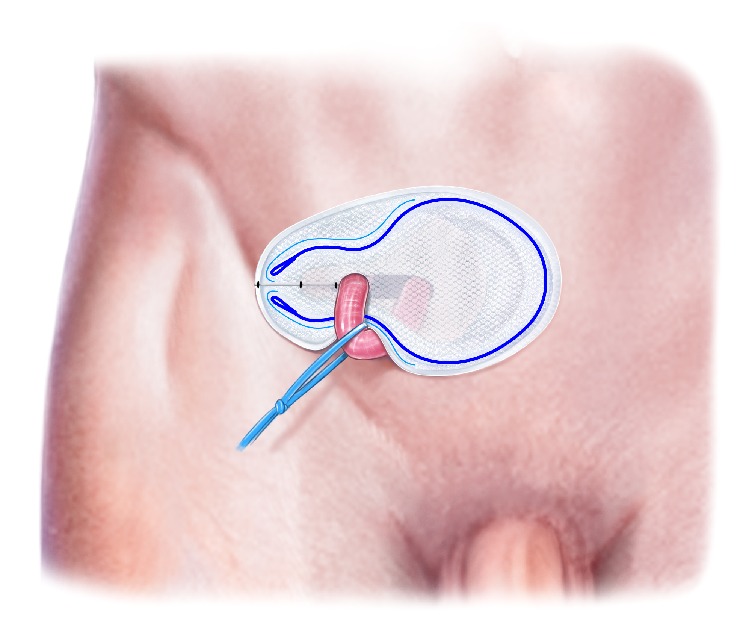
The two tails in the Onflex mesh are closed with nonabsorbable interrupted sutures.

**Figure 11:**
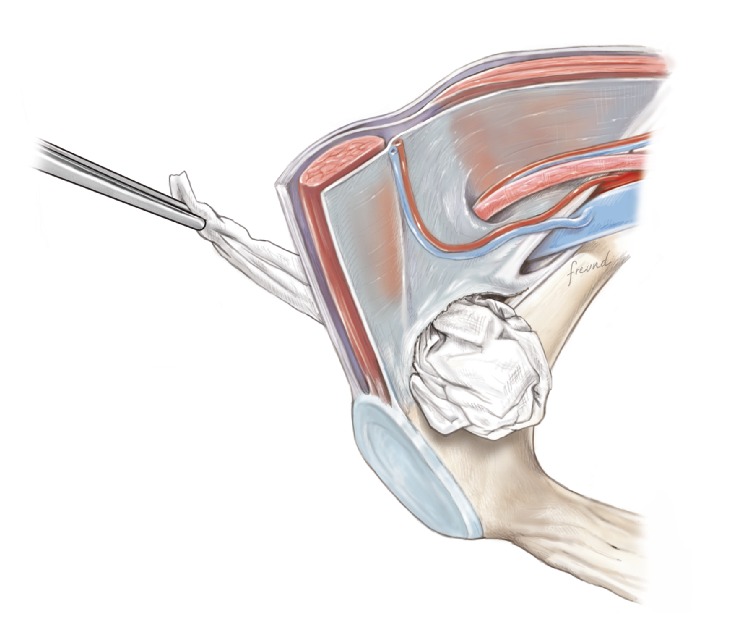
The gauze is removed.

**Figure 12:**
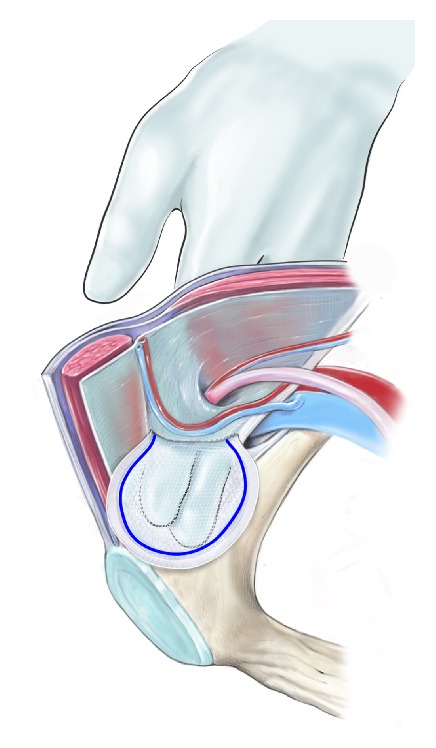
The mesh is placed medially in the preperitoneal space.

**Figure 13:**
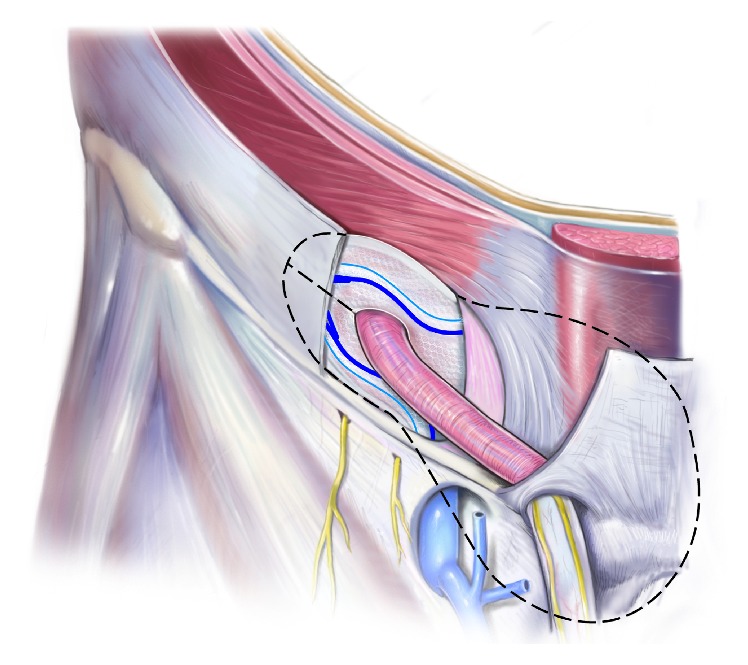
The lateral part of the mesh is placed in the space between the internal and external aponeurosis, and the medial part of the mesh is positioned in the preperitoneal space.

**Figure 14:**
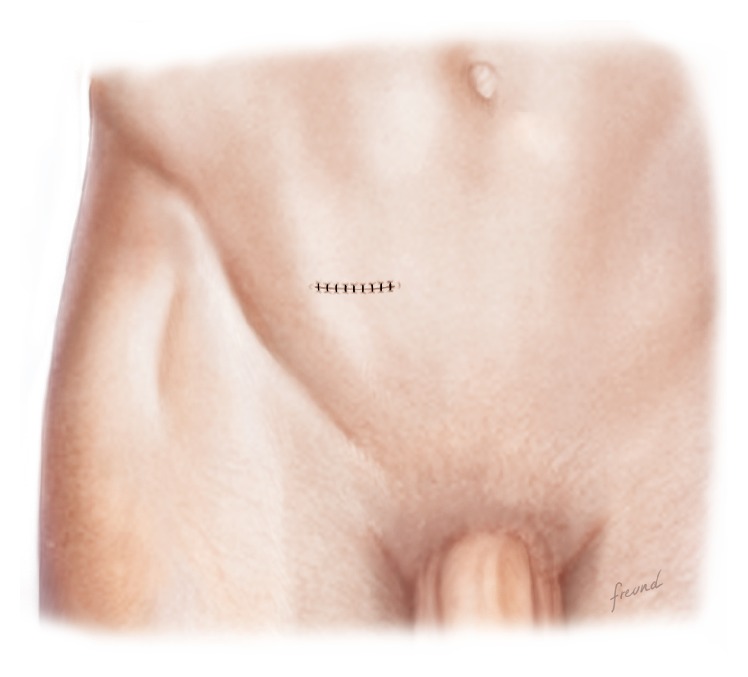
The external fascia is closed with sutures and the skin is closed with sutures or staples.

The procedure is conducted as follows:

- Mark an incision line from the intersection of the index fingers placed superior and lateral to the midpoint of the pubic symphysis to a point 4 cm horizontal and lateral to this intersection point (Figure 1). Note that proper determination of the incision site will ensure that the right tissue plane will be created for optimal visualization and prosthetic placement and the superficial anterior branches of the iliohypogastric and ilioinguinal nerves are avoided and spared from injury.
- Incise the marked line down to the level of the subcutaneous tissue.
- Cauterize the superficial epigastric vein as the subcutaneous tissue is dissected to the width of the skin incision exposing the anterior surface of Scarpa's fascia (Figure 2). Carefully dissect Scarpa's fascia with electrocautery until the anterior surface of the external oblique aponeurosis is exposed.
- Begin the dissection of the external oblique aponeurosis by first making a small incision to expose the underlying internal oblique aponeurosis (Figure 3). Then, continue the dissection using forceps and scissors taking care not to injure the internal oblique aponeurosis. The incision in the external oblique aponeurosis should be transversal and not follow the fibers.
- Digitally dissect the space between the external and internal aponeurosis sweeping laterally and superiorly up towards the superior iliac spine to create a dissected tissue plane that will subsequently accommodate the tails of the prosthesis (Figure 4). The dissection should be performed gently and only using the index finger. The finger moves close to the roof of this dissected space, thus again avoiding the nerves that will be positioned on the floor of this newly developed space. The finger will move under the roof (the external aponeurosis) caudally down to meet the pubic bone, where pecten and Cooper's ligament covered by the transversalis fascia are identified with the finger.
- The next step is to isolate and mobilize the spermatic cord. The cord is elevated up and out of the incision site (Figure 5). To facilitate grabbing of the cord, the finger should go along the roof of the newly created space and bend the finger forward in order to get the cord. At this point it is easy to see if there is a medial hernia and if a lateral hernia is present this is left in the spermatic cord until later.
- Digitally explore the transversalis fascia down to the pubic bone and obliquely plunge down through the fascia with the direction towards the bladder (Figure 6). The place for entering the preperitoneal space should be just along the lateral edge of the rectus muscle and just cranial to Cooper's ligament, thus as medial and caudal as possible in the operative field. This is to avoid a conflict with the iliac vessels. Be careful not to make the perforation too large as this will increase the risk of mesh displacement and thereby the risk of hernia recurrence. At this point of the procedure it is important to slow down and to move very gently with the finger in the preperitoneal space. It is not a good idea to dissect digitally in the preperitoneal space as the space for the mesh will be dissected automatically by the gauze placement (see below). If blood is seen coming up from the preperitoneal space there is probably a lesion in the small blood vessels and it is important to look carefully for this and take care of it by electrocautery under direct visual guidance.
- A sterile gauze, preferably size 30 × 40 cm, is placed through the incision into the preperitoneal space and guided down towards the pubic bone to bluntly dissect the space required for insertion of the prosthesis (Figure 7). The direction of the gauze insertion into the preperitoneal space should again be medial and not lateral. It will be easier to maneuver the gauze if it is moist with saline before placement.
- Now the gauze is left in the preperitoneal space for some minutes while a possible indirect hernia component is taken care of. This means that the spermatic cord should be dissected and a hernia sac isolated and taken care of. The Onstep technique does not decide the method of repair of a lateral hernia component so either dissect and invaginate the sac entirely or open and transect the sac according to local routines.
- Cut a slit in the prosthetic patch between the interrupted ends of the memory recoil ring down to the apex of the curved notch of the patch (Figure 8). Be careful not to cut the ring or to open the pocket in the mesh with the ring. Also the lateral parts of the Onflex mesh are removed (Figure 9). When cutting the lateral part, make sure to avoid cutting in the stitched line because it will open the pocket holding the stiff ring in the mesh.
- Place the caudal tail of the patch under the elevated cord. Place three interrupted sutures with nonabsorbable suture material to join the prosthetic tails together (Figure 10). One suture is placed close to the spermatic cord, one at the end of the lateral tails of the patch ensuring that they do not overlap, and one at the midpoint of the slit.
- Now the gauze is removed from the preperitoneal space (Figure 11) and the index finger can gently push the fatty tissue cranially making better room for the mesh.
- The index finger of the other hand is placed in the mesh pocket and the mesh is thereby pushed down to the preperitoneal space (Figure 12). If the finger is not long enough, then a blunt instrument can be used in the pocket instead of your finger.
- Digitally explore (gently) and smoothen the patch ensuring that the patch is fully deployed under the pubic bone into the space of Retzius with no wrinkles or buckles. If wrinkles or buckles are observed or felt this is an indication that the dissected space was insufficient for the size of the patch and further gentle dissection is required. This is typically done by pushing the fatty tissue with the index finger away from the area below the mesh so that the mesh will be placed in close contact with the bone.
- Insert the lateral tails of the prosthetic patch into the previously dissected space (Figure 13) between the external and internal oblique aponeurosis and digitally explore and smoothen to ensure a prober placement.
- Close the external oblique aponeurosis with the suture type and technique of choice, typically absorbable sutures with a running suturing technique. There is no need to close Scarpa's fascia or to apply subcutaneous sutures.
- Inject local anesthetic of choice as appropriate; typically some mL is given below the external aponeurosis and the rest is given by intracutaneous infiltration around the skin incision.
- Close skin with sutures or clips as appropriate (Figure 14).

## 3. Technical Tips

There are certain technical tips available for the Onstep procedure in order to make it easier to perform.

When performing training sessions with the Onstep procedure we usually get questions regarding the placement of the skin incision (Figure 1). People may wonder why the incision is placed more cranially than they are used to when performing the Lichtenstein procedure. The main reason for the more cranial placement of the incision is that the entry into the space between the external and internal oblique aponeurosis is easier because the two aponeuroses at this level are divided into distinct different layers. This makes it easier to get into the correct space. Furthermore, and perhaps most importantly, the entry into the space between the two aponeuroses is cranial to the natural course of the ilioinguinal as well as the iliohypogastric nerves which are running more caudal to the incision site. When the incision in the external aponeurosis is performed, then the dissection after this is done by blunt finger dissection without any instruments. This will hopefully minimize the risk of nerve damage.

In the next part of the procedure the cord has to be mobilized (Figure 5). This can sometimes be a little difficult and the easiest approach may be to use your index finger as a hook and try to take the cord from the caudal part and then lift it up cranially and out of the skin incision. During this part of the operative procedure it is usual to apply some tension/traction on the cord structures at least during the mobilization. This is probably part of the reason why use of local anesthesia infiltration as the sole anesthetic agent may be inadequate to obtain full pain relief during the operation.

During the next step where a perforation has to be made in the floor of the inguinal canal (Figure 6) it is very important to stretch out the tissue with the index finger. Especially when a medial hernia is present the tissue may be quite floppy and if this is the case then it is more difficult to stretch the back wall of the inguinal canal with the index finger. Here it may be necessary sometimes to use a gauze on the index finger in order to apply more force during the stretching procedure.

When the back wall of the inguinal canal is stretched as much as possible, the index finger of the other hand is used to plunge on the pubic bone/coopers ligament. The place to make the perforation should be chosen as close as possible to the lateral edge of the rectus muscle and as close as possible to the pubic bone. It is important to make the perforation in the back wall of the inguinal canal not too big. This means in detail that as soon as your index finger has gone through the tissue here then the speed of the operation has to be lowered significantly. It is important to move the index finger in the preperitoneal space with extreme caution and very slowly. Push the fatty tissue in the preperitoneal space in the cranial direction as gently as possible thus moving it away from the pubic bone. Be aware though that there are small vessels in this area, which may be destroyed even by gentle movement of the finger. So, in order to avoid bleeding, be really careful and do not move the finger around too much. If there is bleeding, put in a proper sized speculum and then under direct visualization take care of the problem with electrocautery.

Another important problem with the perforation is not to make it too big. If the perforation is too big, then the mesh will be able to slide up and down and then it will be possible for the mesh to shift its position resulting in a recurrence. On the other hand the perforation should not be too small because then the mesh will fold when passing through the perforation. So, a good advice is to do the perforation with the index finger and be very gentle with the finger movement, and then the end result of the size of the perforation is probably around 1.5 fingers in width.

During the next step where a gauze is placed in the preperitoneal space (Figure 7), it will make the procedure easier if the gauze is moist with isotonic saline. The size of the gauze is also of importance and it is advisable to use a size of at least 30 × 30 cm and preferably 30 × 40 cm.

In rare cases, where, for example, radiation therapy has been used in this area for prostatic cancer, there may be extensive fibrosis, so that dissection in the preperitoneal space will be difficult. Often after radical prostatectomy with radiation therapy it is possible to do an Onstep procedure without any problems at all. However, in rare cases the fibrosis may be extensive so that it may be difficult (and therefore dangerous) to make the preperitoneal dissection in order for the mesh to be placed properly. In these cases simply back up at this point of the operation and do a Lichtenstein procedure instead. The only difference now will be that the skin incision will probably have to be prolonged 1-2 cm laterally in order to make space for the Lichtenstein procedure.

The next part of the operation is placement of the mesh in the preperitoneal space. For this there is a pocket in the mesh, which is intended for the index finger to push the mesh down into the preperitoneal space. However, with short fingers like ours, it may not be possible in the average patient to reach all the way down to the bottom of the mesh placement with the index finger. In these cases it can be easy to use, for example, the handle of the Langenbeck retractor as an extension of the index finger to push the mesh down to its proper place. As always when moving in the preperitoneal space it has to be slow and careful movement. After mesh placement the position is checked by the index finger on the cranial side of the mesh, so that the mesh placement is felt exactly on the pubic bone without interpositioned fatty tissue. If there is any fatty tissue between the mesh and the bone take care of that so the mesh is positioned directly in the bone.

When the slit in the mesh is closed by sutures it is important to use nonabsorbable sutures instead of absorbable sutures. We have had recurrences because of breakage of the absorbable sutures.

Closing of the skin is the last part of the procedure, but the only thing the patient will see. The use of clips can cause discomfort for the patient in the first days, since the incision is placed right under the belt. Therefore we advise using sutures or if using clips we then advice to keep the bandage on for 3–5 days.

## 4. Potential Concern

The technique does not leave the preperitoneal or the inguinal canal untouched, so concern has been raised of how to manage recurrences. In clinical practice this is however not a problem. Recurrences may be managed by a re-Onstep with dissection in the plane superficial to the old mesh and then placing the mesh between the old mesh and the public bone. Recurrence after Onstep may also be managed by laparoscopic technique.

Another concern may be how to manage large scrotal hernias with the Onstep technique. A large scrotal hernia is difficult to mobilize regardless of surgical technique, and in our experience it has not been more difficult with the Onstep as it would have been with a standard Lichtenstein approach even if the hernia is irreducible before operation.

Patients with previous prostate cancer who have received radiation therapy as well as radical prostatectomy may present with a symptomatic inguinal hernia and these patients can be offered an Onstep operation. Most often there will be no technical surgical problems, but if the preperitoneal space presents with excessive fibrosis, you can always convert to a Lichtenstein operation with just a slight extension of the incision.

## 5. Conclusion

We have presented a thorough technical description of the Onstep procedure together with tips and tricks. This paper can be used for guidance both before and after training of the technique and to refresh the steps. Proper hands-on training should always be conducted, supervised by an experienced Onstep surgeon, before conducting this procedure.
